# The Dutch cutaneous squamous cell carcinoma and metastasis (D-SQUAME) study: a nationwide discovery cohort and nationwide validation cohort with nested case–control designs for risk prediction modeling

**DOI:** 10.1007/s10654-026-01384-4

**Published:** 2026-03-12

**Authors:** Olivia F. M. Steijlen, Barbara Rentroia-Pacheco, Antien L. Mooyaart, Catherine Chia, Marieke W. J. Louwman, Quirinus J. M. Voorham, Annette H. Bruggink, Tamar E. C. Nijsten, Marlies Wakkee, Loes M. Hollestein

**Affiliations:** 1 Department of Dermatology, Erasmus MC Cancer Institute, University Medical Center Rotterdam, Rotterdam, The Netherlands; 2 Department of Pathology, Erasmus University Medical Center, Rotterdam, The Netherlands; 3 Department of Research and Development, Netherlands Comprehensive Cancer Organization, Utrecht, The Netherlands; 4 Dutch Nationwide Pathology Databank (Palga), Houten, The Netherlands; 5 Department of Pathology, Research Institute for Medical Innovation, Radboud University Medical Center, Nijmegen, The Netherlands

**Keywords:** Cutaneous squamous cell carcinoma, Metastasis, Risk prediction, Biomarkers, Multi-omics, Digital pathology, Multimodal data, Population-based, Prognostic

## Abstract

Current clinical risk stratification systems are limited in their predictive value for metastasis in cutaneous squamous cell carcinoma (CSCC). Progress requires large population-based datasets integrating clinical, pathological, and molecular data and efficient study design, because the volume of patients is large and the event rate is low. This study describes two nationwide cohorts and the collection of CSCC samples with long-term follow-up and sufficient metastatic events to enable prognostic modelling. Linked data of the Netherlands Cancer Registry and the Dutch Nationwide Pathology Databank were used to collect two nationwide CSCC cohorts and perform nested case–control studies. In the discovery cohort (first CSCC diagnosis: 2007–2009), each metastatic case was matched to a non-metastatic control with similar metastatic risk. In the validation cohort (first CSCC diagnosis: 2017–2018), each case was matched to both a random and a risk-matched control. Tissue sections were prepared for hematoxylin and eosin staining, RNA/DNA sequencing, and immunostaining. The discovery cohort included 19,120 CSCC patients with ten years of follow-up and 472 samples (236 case–control sets) with a median time to metastasis of 1.1 (IQR 0.5–2.1) years. The validation cohort included 25,921 CSCC patients with at least five years of follow-up and 555 samples (~ 185 sets with 2 types of controls, with a median time to metastasis of 0.70 (0.3–1.5) years. This design enables the development of absolute risk prognostic models with sufficient number of events. Clinical, histopathological, and molecular data can be combined, paving the way towards more personalized treatments for CSCC patients.

**Supplementary Information:**

The online version contains supplementary material available at 10.1007/s10654-026-01384-4.

## Introduction

Cutaneous squamous cell carcinoma (CSCC) is one of the most prevalent cancers worldwide, with a rising incidence that poses significant clinical and public health challenges [1, 2]. Despite its growing burden, CSCC remains difficult to study due to major epidemiological and methodological hurdles.

The sheer volume of tumors diagnosed and the frequent perception of CSCC as a relatively non-aggressive malignancy, contributes to the under-registration and sometimes omission of CSCC in cancer registries. In some registries, only the first diagnosis per patient or the first CSCC per patient per year is registered [2–6]. Information on disease progression, such as metastasis, has rarely been routinely recorded by cancer registries [7, 8]. In case progression is recorded in single or multicenter studies, it is often missing or inconsistent, as the primary CSCC may not have been diagnosed in the same center as the metastasis. As a result, follow-up data are difficult to collect and tumor samples are hard to obtain. Difficulties in effectively retrieving large and representative CSCC cohorts have limited molecular research in CSCC. As a result, only a few single center studies have investigated the molecular characteristics of CSCC [9, 10].

Metastasis in CSCC is rare, with a cumulative incidence of approximately 2% [5, 11]. Consequently, identifying a sufficient number of metastatic events requires the inclusion of thousands of patients. Studies of this size are difficult to combine with detailed molecular analyses, because collecting samples and performing expensive laboratory techniques is often not feasible. A nested case–control (NCC) design offers a practical solution, as it allows detailed molecular analyses to be performed on metastatic cases and (matched) controls within a large population-based cohort, while still being able to estimate absolute risk [12]. This design is particularly suitable for developing and validating models that predict metastasis risk.

Using this design, the Erasmus MC (EMC) model was developed based on nationwide data to predict individual metastasis risk based on clinicopathological features [13]. This absolute risk model outperformed the established American Joint Committee of Cancer (AJCC) and Brigham and Women’s Hospital (BWH) staging systems. This is particularly relevant because many earlier studies could only estimate relative risk, which is a comparison between groups, whereas absolute risk provides the actual probability that a metastatic event will occur in a specific population and is therefore more clinically informative [12].

The EMC model shows great potential for assisting decisions on follow-up and adjuvant therapy [13]. However, the predictive ability of the EMC model is likely to be further enhanced by integrating molecular data, including gene expression profiles and digital pathology analysis. This may help identify aggressive tumors not evident from conventional clinical and pathological risk factors.

Due to aforementioned hurdles in CSCC research, there are no large-scale molecular studies in CSCC. Even widely used genomic databases like The Cancer Genome Atlas (TCGA), which changed our understanding of several cancer types, do not include any molecular data of CSCC [14]. To overcome this hurdles, we designed the D-SQUAME study and created a nationwide discovery cohort and a nationwide validation cohort with extensive follow-up and a sufficient number of metastatic events for risk prediction modelling. By using a nested case–control design and the linkage between the cancer registry and the nationwide pathology database, we were able to retrieve the primary CSCC that caused the metastasis and generate molecular data of these tumors. Here we describe the design of the D-SQUAME study. The generated multi-modal data will be used and integrated in future work to develop and validate accurate risk prediction models capable of identifying CSCCs at high risk for developing metastases.

## Methods

### Data sources

This study leveraged national cancer, pathology, and organ transplant registries in the Netherlands to identify patients with CSCC, assess disease progression through histopathologic confirmation of metastasis, and determine immunocompromised status respectively.

The discovery cohort included all newly diagnosed CSCC patients in the Netherlands from 2007 to 2009 with more than 10 years of follow-up. The validation cohort included all newly diagnosed CSCC patients in 2017 and 2018 with at least 5 years of follow-up. CSCC in the Netherlands is routinely registered by the Netherlands Cancer Registry (NCR) [15]. This registry is embedded in the Netherlands Comprehensive Cancer Organization (IKNL). The NCR records newly diagnosed malignancies since 1989. Since 2016, the NCR has used automated notifications from the Dutch Nationwide Pathology Databank (Palga) and therefore includes all first primary and subsequent CSCC per patient [16]. Before 2016, trained data managers from NCR collected data from pathology reports and digital patient records of each first CSCC per patient per year. From mid-2016 onwards, CSCC are automatically imported from Palga without manual registration [5]. Invasive CSCCs are identified using the International Classification of Diseases for Oncology, third edition (ICD-O-3) morphology codes 8010/3, 8051/3, 8070/3–8076/3, 8078/3, 8081/3, 8083/3, and 8084/3, and topography code C44 for skin [17]. Extracted variables included sex, age at diagnosis, year of diagnosis, TNM stage, and vital status. We also requested information on hematologic malignancies (HMs) from NCR, including type and date of diagnosis (supplementary material “S1 Variables requested from the Netherlands Cancer Registry (NCR)”), as they provide valuable insight into immune status.

Palga, established in 1971, is a national pathology databank covering all pathology laboratories in the Netherlands. It comprises a decentralized information system, central databank, and a communication infrastructure [16]. Pathology excerpts are transferred to the central databank and include encrypted patient identifiers and demographic data. Diagnoses are structured and coded based on topography, morphology, function, procedure, and disease.

Because CSCCs were only recorded at first primary diagnosis in the NCR up to 2016, and patients may develop multiple CSCCs, we linked NCR data with Palga to capture all subsequent primary tumors and disease progression. Using retrieval terms on CSCC, metastasis, and lymph node tissue, we collected all CSCC-related pathology reports of patients identified by NCR. These included clinical information, macroscopy, microscopy, and diagnostic conclusions. Detailed information on the retrieval terms can be found in the supplementary file “S2 Codelists”. To distinguish new primary CSCCs from recurrences, we applied a rule-based algorithm using ICD-O-3 anatomical subsites, lateralization, and a 3-month time window [18].

Data on solid organ transplant recipients (SOTRs) were obtained through linkage with the Netherlands Organ Transplant Registry (NOTR) from the Dutch Transplantation Foundation [19]. The NOTR is a nationwide database established in 2004 that collects data on all organ transplant recipients in the Netherlands, including retrospective data on transplantations performed prior to its establishment. From the NOTR, we retrieved the date of transplantation and the type of transplanted organ. Patients were classified as immunocompromised if they had a history of hematologic malignancy (HM) or were SOTRs at the time of cutaneous squamous cell carcinoma (CSCC) diagnosis.

The Dutch National Tissue Portal (DNTP) was used to request the selected tumor material [20]. The DNTP utilizes Palga’s nationwide network and facilitates the use of tissue samples for research. The DNTP consists of a centrally organized internet portal to request (tumor) tissue material. Dedicated employees provide practical support at peripheral pathology departments in the Netherlands.

The use of left-over diagnostic tissue samples for scientific research is based on the ‘opt-out’ principle, as stated in the Code of Conduct for Health Research from the Committee on Regulation of Health Research. Consequently, a waiver of informed consent was granted by the ethical committee of the Erasmus MC (MEC-2020–0147).

### Study design

**Fig. 1:**
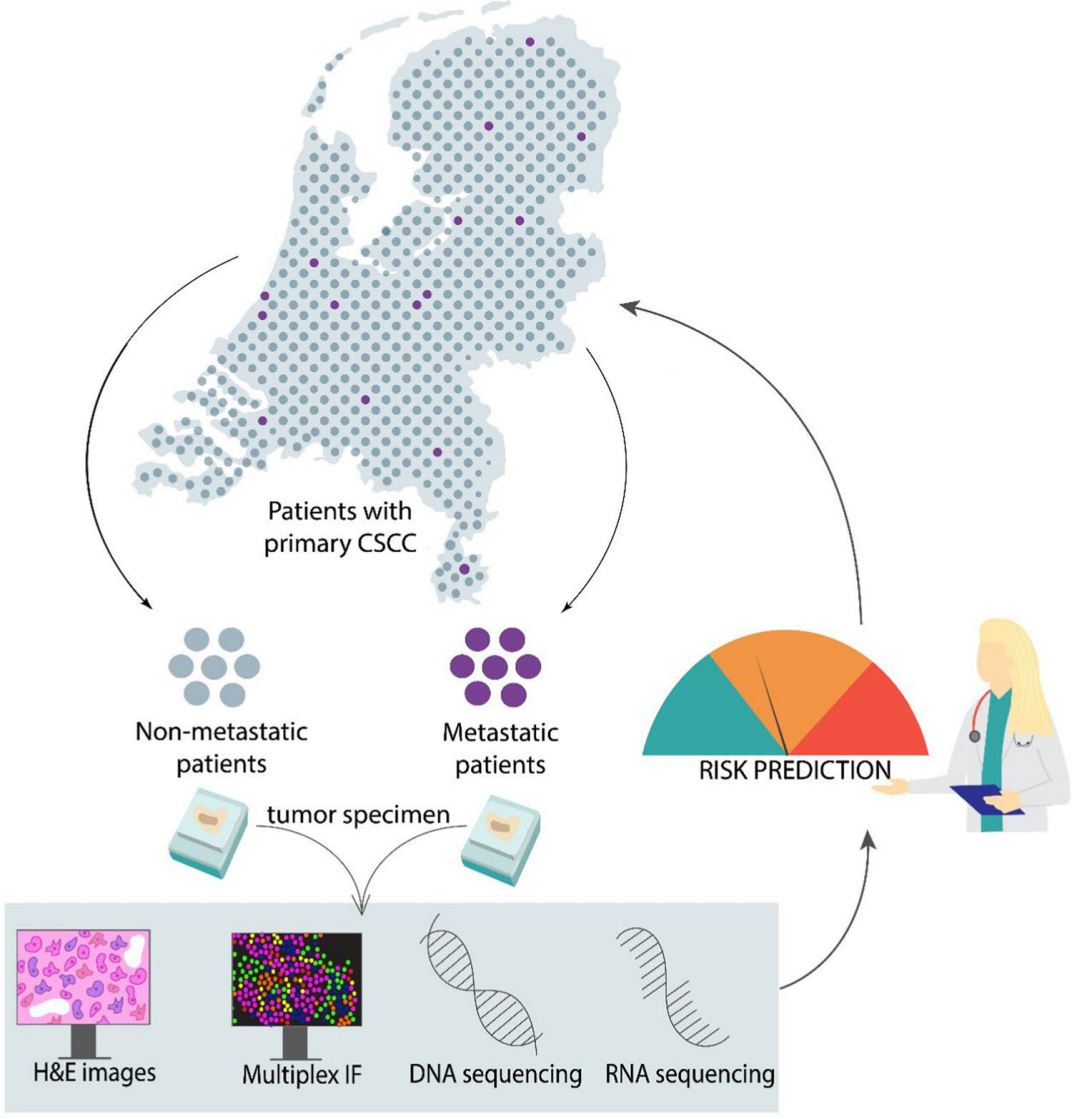
Nested case–control design in nationwide cohort of primary CSCC patients in the Netherlands. (Abbreviations: *CSCC* cutaneous squamous cell carcinoma, *H&E* Hematoxylin and Eosin staining, *IF* immunofluorescence)

We selected two nationwide cohorts, the discovery and validation cohort, and performed two nested-case control (NCC) studies (Fig. 1).

### Discovery cohort

The discovery cohort includes patients with a first CSCC between 1 January 2007 and 31 December 2009, with follow-up until 30 March 2022 including complete pathological histories. From this cohort, we identified metastatic cases and non-metastatic controls (the definition of cases is described in the *Identification of metastasis* section). We extracted all prognostic variables from the pathology reports using a rule-based algorithm based on free text recognition and specific Palga codes (supplementary file “S2 Codelists”) [21].We aimed to identify novel (molecular) prognostic factors that are independent of the already well-established prognostic factors that are included in the EMC model. Therefore, controls were matched to cases based on similar predicted 5-year metastatic risk based on the EMC model, follow-up time, type of surgical procedure and pathology lab (Table 1). We aimed for the closest possible risk match between cases and controls; a difference of up to 10% was accepted only when a more exact match was not available. All extracted variables of the matched cases and controls were manually reviewed to ensure accurate variable selection. If controls were incorrectly matched due to errors in variable selection, a new control was selected until each case was paired with a suitable control.

**Table 1:** Variables used for matching

Variable	Matching approach
Follow-up time	Required: control must have longer follow-up than the matched case
Type of surgical procedure	Required exact match (e.g., biopsy, excision (WLE/MMS/PMMS), or both)^a^
EMC-model^b^ risk score	Loosely matched: allowed within a ± 10% range
Pathology laboratory	Loosely matched: ignored only if all other variables matched but lab was unavailable^c^

^a^We matched cases and controls on the type of surgical procedure to avoid confounding effects related to wound healing after surgical procedures and differences in relative proportions of tumor/stromal content due to procedural differences

^b^The Erasmus MC model is a Cox regression model that provides an individual-level absolute risk estimate for metastasis [8]

^c^Matching by pathology laboratory was not always feasible due to few available tumors in some labs and was primarily applied for logistical reasons

*Abbreviations: WLE* Wide Local Excision, *MMS* Mohs Micrographic Surgery, *PMMS* Paraffin-embedded margin-controlled micrographic surgery

### Validation cohort

The validation cohort includes patients with a first CSCC between 1 January 2017 and 31 December 2018 with follow-up until 8 June 2023 including complete pathological histories. Pathology reports for all cases and potential controls were reviewed and selected. Again, all relevant variables were extracted from the pathology reports using the rule-based algorithm.

To be able to validate novel prognostic factors or models, we constructed a validation cohort using both a matched nested case–control design and a nested case–control design with randomly selected controls. This enables the validation of the additive prognostic value of the newly identified molecular features in addition to the EMC model (using the matched design) and the exploration of their standalone prognostic value (using the random design). This dual selection of controls will allow the exploration of risk thresholds relevant to specific clinical applications and the identification of the optimal combination of the EMC model and molecular features. For the matched design, the metastatic cases were matched to non-metastatic controls based on predicted 5-year metastatic risk based on the EMC model, replicating the design of the discovery cohort (Table 1). This resulted in a risk-matched NCC dataset, allowing validation within a comparable setting. For the design with randomly selected controls, the same cases were matched to non-metastatic controls selected without matching on predicted risk. Controls were matched on pathology lab (to facilitate the retrieval of the samples), follow-up time (to ensure incidence density sampling) and type of surgical procedure (biopsy or excision).

### Identification of metastasis

The primary outcome of this study was the occurrence of metastasis caused by CSCC. Metastasis is defined as the presence of CSCC cells in the skin, the first draining lymph nodes or distant organs (including skin metastasis) outside the primary tumor site.

Metastasis of CSCC most commonly occurs within the first year following diagnosis of a primary tumor [5]. Metastases detected within four weeks of the initial CSCC diagnosis, or those identified during the planned diagnostic work-up at the time of primary diagnosis (including ultrasound and biopsy of suspicious lesions), were classified as metastases at baseline.

The registration of metastasis includes patients with histopathologically confirmed metastasis, as this information was retrieved from pathology reports. Potential metastases were retrieved using a rule-based algorithm with a free-text search and Palga codes confirming or suggestive of metastasis [21]. These records were manually read by clinical researchers, and uncertainties concerning the presence of metastasis or primary origin were discussed within the lead dermatologist (M. Wakkee) to reach consensus.

The process for identifying the culprit tumor responsible for metastasis was mainly based on the location of both the metastasis as the primary tumor. In patients with multiple CSCCs, it can be difficult to determine which tumor caused the metastasis. To make this decision, we considered three factors: (1) the location of the primary tumor relative to the metastatic lymph node (e.g., if metastasis occurred in the right axilla, we considered the CSCC on the right arm more likely to be the cause than one on the right leg); (2) the time between the development of the CSCC and the detection of metastasis, with tumors that occurred within 2 years being more likely to be the source than those that occurred over 5–10 years earlier; and (3) whether the potential primary tumor had high-risk features. Since we focused on risk factors, the third criterion was used mainly to support decisions made using the first two. Cases where we could not confidently identify the primary tumor using these criteria were excluded due to an unknown primary origin.

### Sample processing

We obtained Formalin-Fixed Paraffin-Embedded (FFPE) tumor specimens, corresponding Hematoxylin and Eosin-stained (H&E) slides when available, as well as the anonymized pathology reports. Tumor material from the initial procedure of the primary tumor was used. If both biopsy and excision were performed, we requested both specimens. The initial procedure was prioritized to obtain tumor material before wound healing occurred, thereby avoiding potentially overpowering gene expression analyses with wound healing-related transcriptional activity [22]. The excision specimen was additionally required to enable pathological revision, as key characteristics such as depth of invasion and differentiation grade cannot be reliably assessed from biopsy material alone.

Including multiple procedures, such as biopsy, wide local excision (WLE) and Mohs micrographic surgery (MMS) or paraffin-embedded margin-controlled micrographic surgery (PMMS) captures biological and histological heterogeneity and accounts for potential prognostic differences between surgical approaches.

After reviewing the original H&E slides, we selected the FFPE block containing the largest area of intact, non-necrotic tumor tissue for further processing. When multiple blocks were available, the middle block was prioritized because it typically included the largest tumor area and deepest invasive front of the tumor. Each block was sectioned to prepare a new H&E slide, which was digitized. A dermatopathologist reviewed the digitized slides to verify the availability of sufficient tumor specimen for downstream analyses. Tumors were excluded if the selected FFPE block did not contain CSCC tissue or if the H&E slide of the sample could not be reassessed due to technical issues such as FFPE block damage.

**Fig. 2:**
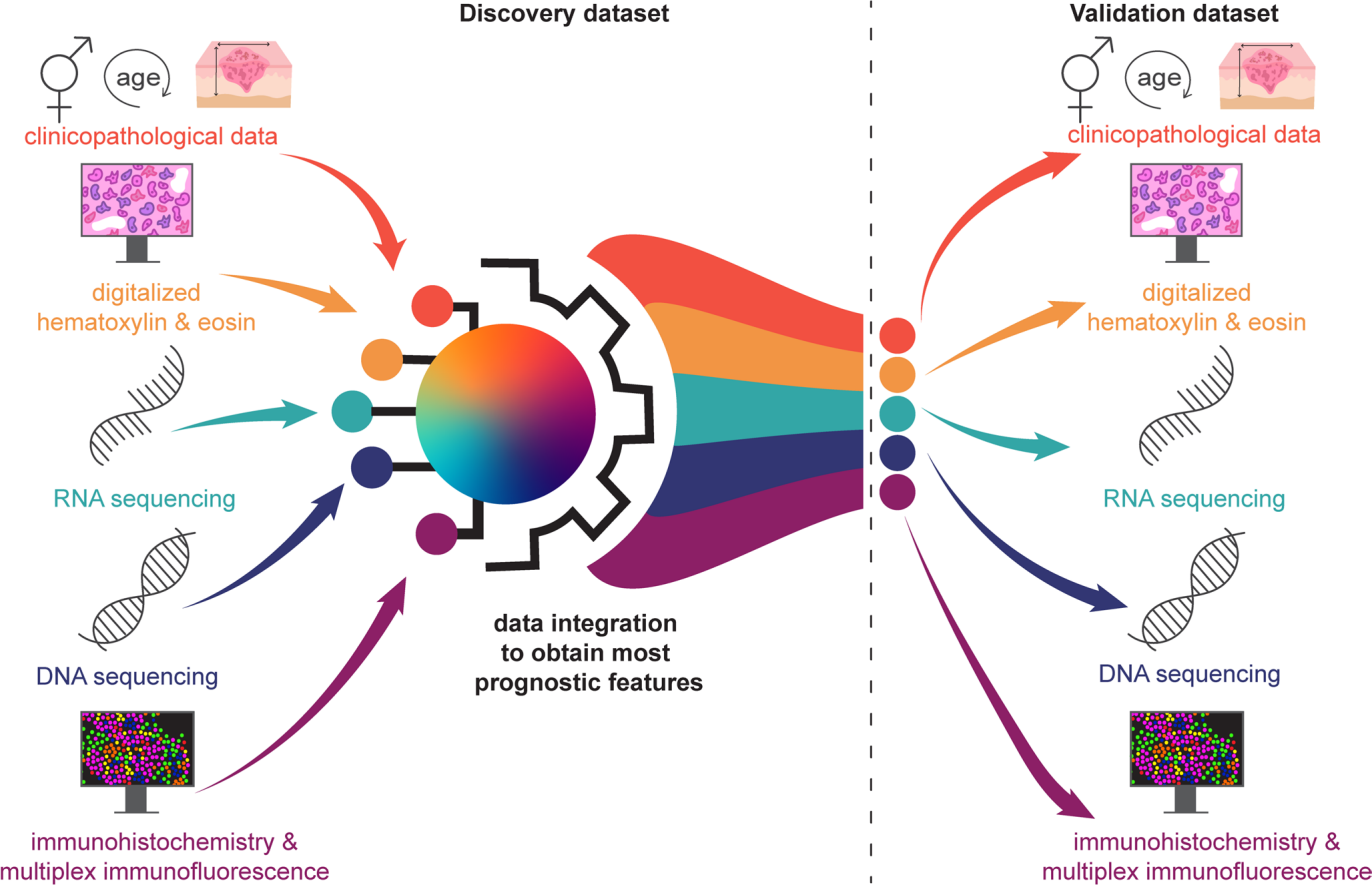
Clinical, imaging, RNA sequencing and DNA sequencing data derived from the discovery dataset will be integrated, and the most prognostic features will be validated in the validation dataset. (Adapted from Zhou et al. [51], used under CC BY 4.0 license)

To be able to develop prognostic models integrating multi-omics, imaging, and clinical data, we cut slides from each sample for H&E staining, RNA sequencing, DNA sequencing and immunohistochemistry (IHC) or multiplex immunofluorescence (IF) from both the samples of the discovery and validation NCC datasets (Fig. 2). Each FFPE block yielded 32 4 µm sections: 5 for H&E staining, 10 for DNA sequencing, 13 for RNA sequencing, and 4 for IHC. The first section was always stained with H&E. We alternated the order of slides for each type of experiment to avoid selecting areas with varying tumor aggressiveness for different type of experiments due to tumor heterogeneity (see supplementary material “S3 Sample processing protocol”). Slides for IHC were dipped in 60 °C paraffin to preserve tissue integrity. RNA was extracted from whole slides to capture RNA from both tumor cells and surrounding immune cells, supporting analysis of the tumor microenvironment. RNA extraction was performed as soon as possible, typically within one week, to limit degradation. For DNA sequencing, macrodissection was performed to identify tumor and normal skin regions and extract DNA from each region separately, allowing identification of somatic genomic alterations. As DNA is more stable than RNA, no specific timing was required for DNA isolation. Detailed protocols for RNA and DNA extraction and sequencing will be described in separate manuscripts.

### Histopathological reassessment

We re-evaluated all histopathological slides to ensure accurate, consistent, and complete data collection. This was necessary because the original pathology reports often lacked important tumor characteristics [23]. The reassessment also allowed us to document additional histological features that are not routinely reported in clinical practice. H&E slides were scanned at 0.25 µm/pixel using the NanoZoomer S360MD Slide Scanner (Hamamatsu Photonics, Japan). The digital slides were reviewed using NDP.view2 software (version 2.9.29, Hamamatsu Photonics, Japan). Excision specimens were prioritized for review, If no excision was available, the biopsy was evaluated.

Revision of the histopathological variables was based on published literature and clinical standards [24–26]. These definitions were discussed with a team of dermatopathologists and are provided in the supplementary material (“S4 Histopathological reassessment”). The digital slides were subsequently revised by these pathologists, blinded to the outcome and initial pathology report. Only the anatomical location of the tumor was provided to support an accurate assessment of invasion depth. Any uncertain cases, including difficulties in assessing the depth of invasion or ambiguity in morphological subtype, were re-reviewed in collaboration with the lead dermatopathologist (ALM) to reach consensus.

### Statistical analyses

The sample size calculation for our discovery cohort was guided by the aim to of developing an RNA gene expression profile. In the absence of known effect sizes or defined molecular characteristics in our study population, we referred to a previously published study that successfully developed a gene expression signature for another type of skin cancer based on 160 patients using a targeted sequencing approach [27]. We increased the sample size to 400 samples (200 cases + 200 controls), because we intended to test all expressed genes and required sufficient number of events for robust model development. The number of events per included variable varies between different contexts, but in general at least 10 events per variable are recommended for accurate prediction modelling [28]. This number of events per variable does require shrinkage of the estimated regression coefficients to prevent overfitting. A sample size with 200 events allows to include 20 degrees of freedom in a regression model, which can all be allocated to new prognostic features, because controls were already matched on known prognostic features based on the EMC model.

The sample size of the validation cohort was based on a simulation study, where the power to detect a decrease in model performance was tested in multiple scenarios [29]. Based on these simulations the authors recommended a minimum of 100 events and 100 non-events for external validation studies. We further increased this sample size to at least 150 events and 150 non-events to be able to detect smaller differences of the intercept and the slope of the calibration curve.

In the current study, we present the descriptive statistics of the clinical characteristics for the discovery cohort, the validation cohort, and the nationwide source cohorts.

Statistical analyses were conducted using SAS® (9.4 M8) and R software (version 4.3.3).

## Results

### Sample collection

**Fig. 3:**
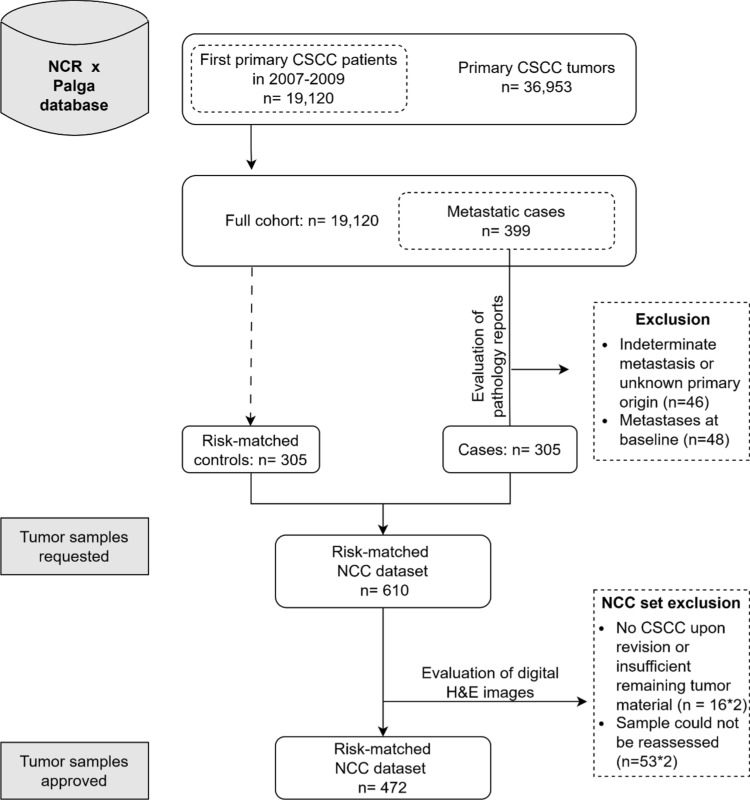
Selection process of the risk-matched case–control sets in the discovery cohort. (Abbreviations: *NCR* Netherlands cancer registry, *EMC* erasmus MC cancer institute, *H&E* hematoxylin & eosin. Risk matched controls: non-metastatic controls were matched to metastatic cases based on absolute metastatic risk calculated by the Erasmus MC model [8])

We identified 19,120 patients with a first primary CSCC diagnosed between 2007 and 2009 from the NCR (Fig. 3). The total number of primary CSCC among these patients was 36,953. During follow-up, we identified 305 histopathologically confirmed metastatic CSCC (1.6%) of which the culprit CSCC could be identified. FFPE tumor blocks were requested for all 305 confirmed case–control sets. A centralized review of the corresponding digitized H&E-stained slides resulted in 236 case–control sets meeting the criteria for further analysis.

**Fig. 4:**
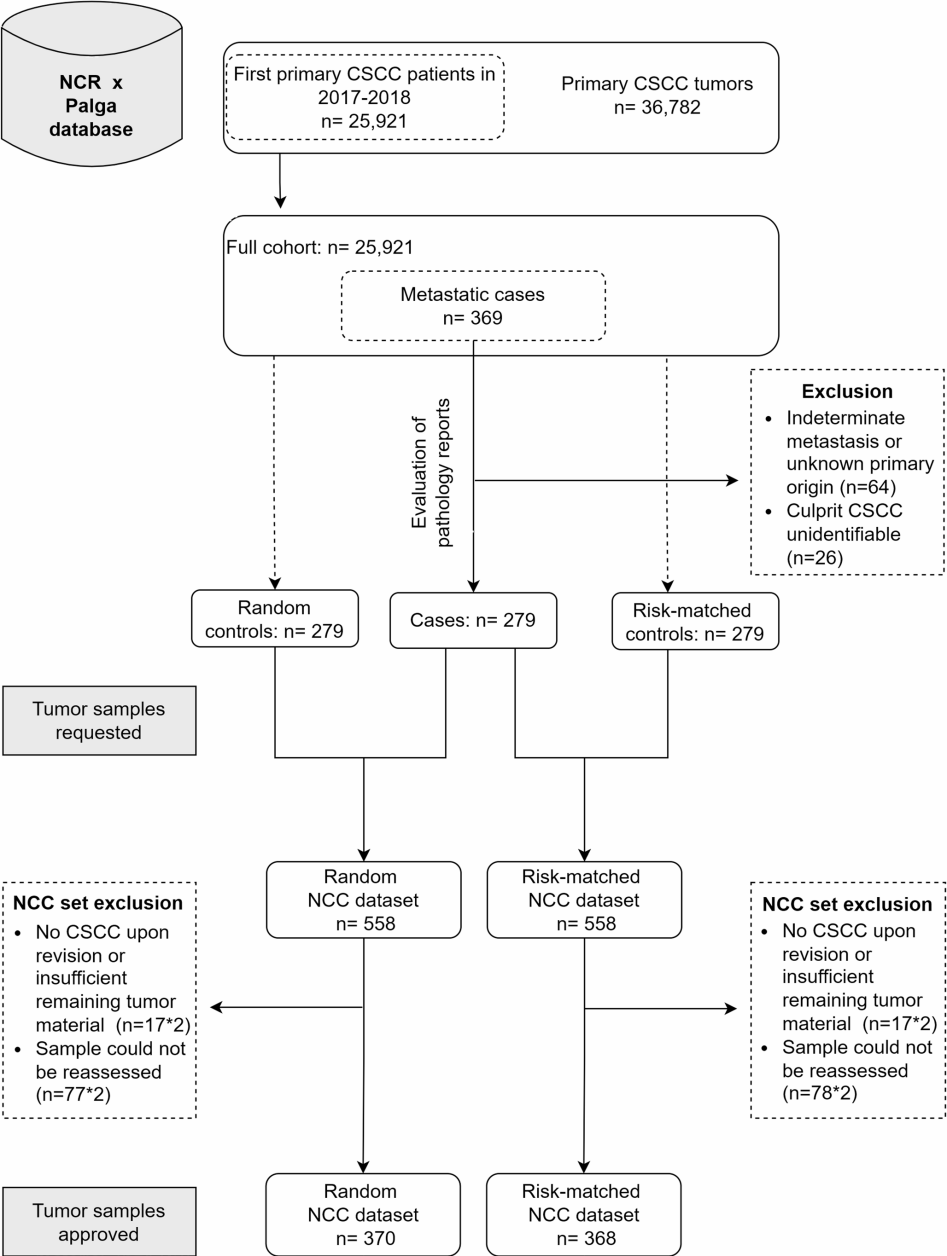
Selection process of the case–control sets (risk-matched and random) in the validation cohort. (Abbreviations: *NCR* Netherlands cancer registry, *H&E* hematoxylin & eosin. Risk matched controls: non-metastatic controls were matched to metastatic cases based on absolute metastatic risk calculated by the Erasmus MC model [8])

For the validation cohort, we identified 25,921 patients diagnosed with a first primary CSCC between 2017 and 2018 (Fig. 4). Among these patients a total of 36,782 primary CSCCs were identified. In the validation cohort, we identified 279 histopathologically confirmed metastatic CSCC (1.1%) of which the culprit CSCC could be identified. Tumor blocks for these 279 case–control sets were requested from pathology laboratories to support both the random and matched NCC study designs. Review of the corresponding digitized H&E-stained slides resulted in 185 random case–control sets and 184 matched case–control sets meeting the criteria for further analysis.

### Clinical characteristics

Table 2 shows the patient characteristics of the discovery and validation NCC datasets and their underlying nationwide cohorts. In the discovery cohort, 57% (n = 10,954) were male. The median age was 76 years (IQR: 67, 83) and the median follow-up time was 8.9 years (IQR: 3.8, 10.4). Five percent of the patients were immunocompromised at the diagnosis of the first primary CSCC (n = 969), either due to a co-occurring hematological malignancy (3.3%) or being an organ transplant recipient (1.8%). The median follow-up time until metastasis was 1.1 years (IQR: 0.5–2.1).

**Table 2:** Patient characteristics of the discovery dataset, the validation dataset and their nationwide source cohorts

		Discovery cohort			Validation cohort			
Characteristics		Nationwide cohort ^a^ (N = 19,120)	Cases (N = 236)	Risk-matched controls (N = 236)	Nationwide cohort^a^ (N = 25,921)	Cases (N = 186)	Controls	
							Risk-matched (N = 184)	Random (N = 185)
Sex, n(%)^b^	Male	10,954 (57)	174 (74)	166 (70)	14,074 (54)	145 (78)	115 (63)	81 (44)
	Female	8,166 (43)	62 (26)	70 (30)	11,848 (46)	41 (22)	69 (38)	104 (56)
Median age (IQR)		76 (67, 83)	78 (72, 84)	78 (73, 84)	76 (67, 83)	77 (71, 83)	81 (75, 87)	79 (72, 84)
Median follow-up until death, or last date of follow-up in years (IQR)		8.9 (3.8, 10.4)	3.4 (1.7, 7.3)	5.2 (3.1, 8.7)	4.6 (4.1, 5.3)	3.1 (1.6, 5.0)	4.3 (2.3, 5.1)	4.29 (2.9, 5.1)
Median follow-up until metastasis in years (IQR)		0.9 (0.4–1.9)	1.1 (0.5, 2.1)	–	1.0 (0.3–2.4)	0.7 (0.3, 1.5)	–	–
Median number of CSCCs per patient (IQR)		1 (1, 2)	2 (1,3)	2 (1,5)	1 (1, 1)	1 (1, 1)	1 (1, 2)	1 (1,2)
1 CSCC, n(%))^b^		12,252 (64)	128 (54)	85 (36)	19,486 (75)	143 (77)	127 (69)	149 (81)
2 CSCCs, n(%)^b^		3538 (19)	44 (19)	41 (17)	4149 (16)	26 (14)	30 (16)	24 (13)
3 CSCCs, n(%)^b^		1412 (7.4)	21 (8.8)	30 (13)	1312 (5.1)	7 (3.8)	20 (11)	9 (4.9)
≥ 4 CSCCs, n(%)^b^		1918 (10)	43 (18)	80 (34)	974 (3.8)	10 (5.4)	7 (3.8)	3 (1.6)
Haematological malignancy, n (%)	At the time of CSCC diagnosis	629 (3.3)	22 (9.3)	16 (6.8)	927 (3.6)	13 (7.0)	11 (6.0)	5 (2.7)
	During follow-up	425 (2.2)	–	–	284 (1.1)		–	–
Solid organ transplant recipient, n (%)	At the time of CSCC diagnosis	340 (1.8)	15 (6.4)	22 (9.3)	327 (1.3)	7(3.8)	5 (2.7)	3 (1.6)
	During follow-up	42 (0.2)	–	–	16 (0.06)	–	–	–

^a^For the full cohorts, characteristics are measured at the diagnosis of first CSCC. ^b^ Percentages may not total 100% due to rounding

*Abbreviations: CSCC Icutaneous squamous cell carcinoma, IQR* interquartile range

The first primary CSCC of the patient registered in NCR (Table 3) was most often located on the face (40%) or the upper extremities (16%). The majority of the registered first primary CSCCs (78%) were T1 tumors staged according to the AJCC.

**Table 3:** Tumor characteristics first primary CSCCs of the full cohort recorded by the Netherlands Cancer Registry (NCR)

Tumor characteristics		Discovery cohort (N = 19,120)	Validation cohort (N = 25,921)
Tumor location, n(%)^a^	Lip	450 (2.4)	348 (1.3)
	Eyelid	217 (1.1)	166 (0.6)
	Ear	1930 (10)	1199 (4.6)
	Face	7616 (40)	7871 (30)
	Scalp and neck	2363 (12)	4327 (17)
	Trunk	1536 (8.0)	2086 (8.1)
	Upper extremities	3090 (16)	4269 (16)
	Lower extremities	1742 (9.1)	2702 (10)
	Overlapping	76 (0.4)	68 (0.3)
	Unknown	100 (0.5)	2885 (11)
AJCC staging^b^ (NCR), n (%)	Tx	2346 (12)	
	T1	14,826 (78)	–
	T2	1037 (5.4)	–
	T3	95 (0.5)	–
	T4	89 (0.5)	–
	Unknown	727 (3.8)	–

^a^Percentages may not total to 100% due to rounding. ^b^ NCR stopped recording the AJCC staging of the first primary CSCC since 2016. This information is not available for the validation cohort

In the validation cohort, 54% of the patients were male (Table 2). The median age was 81 years (IQR: 75, 87), and the median follow-up time was 4.3 years (IQR: 2.3, 5.0). Comparable to the discovery cohort, 5% (n = 1272), of the patients were immunocompromised at the diagnosis of the first primary CSCC. The median follow-up time until metastasis was 0.7 years (IQR: 0.3, 1.5).

In both the NCC dataset of the discovery cohort and the NCC dataset of the validation cohort with the risk-matched controls, there is a clear effect of the matching resulting in inclusion of a higher percentage of males and older patients, and a higher number of CSCCs per patient compared to the nationwide cohort. The randomly selected controls in the validation cohort were more representative of the nationwide cohort based on patient characteristics.

### Primary tumors

Table 4 shows the tumor characteristics from all CSCCs based on the characteristics retrieved from the pathology reports. The most frequent location of CSCC in the nationwide discovery cohort was the face (47%, 17,387/36,953 CSCC). Of all 572 primary CSCCs of the discovery NCC dataset, most CSCC occurred on the face (cases: 68% (n = 160/236), controls: 66%, n = 156/236). A similar pattern was observed in the NCC datasets of the validation cohort (cases: 66% n = 122/186), risk-matched controls: 63%, n = 116/184) with a lower proportion in the random controls (45%, n = 84/185), which was more similar to the nationwide validation cohort (48%).

**Table 4:** Tumor characteristics of the discovery dataset, the validation dataset and their nationwide source cohorts

	Discovery cohort			Validation cohort			
Characteristics	Nationwide cohort^b^ (N = 36,953)	Cases (N = 236)	Controls (N = 236)	Nationwide cohort^b^ (N = 36,782)	Cases (N = 186)	Risk-matched (N = 184)	Random (N = 185)
Tumor location, n(%)							
Trunk and extremities	12,386 (34)	43 (18)	60 (25)	14,225 (39)	32 (17)	51 (28)	71 (39)
Scalp and neck	4446 (12)	31 (13)	20 (8.5)	4,645 (13)	31 (17)	17 (9.2)	28 (15)
Face	17,385 (47)	160 (68)	156 (66)	17,613 (48)	122 (66)	116 (63)	84 (45)
Unknown	2736 (7.4)	2 (0.8%)	0	299 (8)	0	0	2 (1.1 )
AJCC8 staging based on the pathology reports^a^, n (%)							
Known T stage	1,529 (4.1)	–	–	15,104 (41)	–	–	–
Cannot be determined	35,424 (96)	–	–	21,678 (59)	–	–	–
AJCC8 staging rescored by dermatopathologist based on H&E slide, n(%)							
T1	NA	70 (30)	123 (52)	NA	60 (32)	90 (49)	108 (58)
T2	NA	20 (8.5)	37 (16)	NA	23 (12)	24 (13)	18 (9.7)
T3	NA	68 (28)	44 (19)	NA	49 (26)	27 (15)	12 (6.5)
T4	NA	2 (0.8)	1 (0.4)	NA	0	0	0
Unknown	NA	76 (32)	31 (13)	NA	54 (29)	43 (23)	47 (25)
Surgical procedure^c^, n(%)							
Biopsy only^d^	–	*not included*	*not included*	–	13 (7)	11 (6)	13 (7)
WLE/MMS/PMMS only	–	120 (51)	120 (51)	–	87 (47)	87 (47)	86 (47)
Biopsy + WLE/MMS/PMMS	–	116 (49)	116 (49)	–	86 (46)	86 (47)	86 (46)

^a^AJCC staging in this table is based on the 8th edition of the AJCC Cancer Staging Manual

^b^AJCC staging of nationwide cohorts is based on variables extracted from the pathology reports

^c^Surgical procedure data were not available for the nationwide cohort, as this information required manual review to accurately identify the correct procedure

^d^Samples with only a biopsy procedure were not included in the discovery cohort

*Abbreviations: WLE* Wide Local Excision, *MMS* Mohs Micrographic Surgery, *PMMS* Paraffin-embedded margin-controlled micrographic surgery

In 96% (n = 35,424 /36,953)of the pathology reports in the discovery cohort, information on the AJCC8 staging criteria was incomplete, preventing accurate determination of T-stage distribution within this cohort. In the validation cohort, data completeness improved, although information on AJCC8 staging remained missing for 59% (n = 21,678/36,782) of CSCCs. Most CSCCs were classified as T1. 12% (1877 + 7/15,104) of CSCCs with known AJCC8 staging were T3/T4 CSCC.

The majority of the cases were surgically treated with WLE, MMS or PMMS representing 51% (120/236) of cases in the discovery NCC dataset and 46% (86/186) in the validation NCC dataset. In addition, the validation NCC dataset included primary CSCCs managed with biopsy only (7%, 13/186), likely followed by adjuvant radiotherapy, to facilitate validation of the developed molecular biomarkers in these samples.

### Metastasis

Table 5 shows the characteristics of the histopathologically confirmed metastatic cases and reports the anatomical locations of the initial metastatic events and does not include information on subsequent disease progression.

**Table 5:** Characteristics of the histopathologically confirmed metastases of the cases of the discovery and validation NCC datasets

Characteristics metastases		Discovery NCC dataset	Validation NCC dataset
		N = 236	N = 186
Metastasis at baseline^a^, n(%)		*not included*	29 (16)
Metastasis during follow-up, n(%)		236 (100)	157 (84)
Median follow-up until metastasis (years), median (IQR)		1.1 (0.5, 2.1)	0.70 (0.3, 1.5)
Location of first metastasis, n(%)			
Lymph node	Cervical^b^	77 (33)	96 (52)
	Axillary^c^	26 (11)	21 (11)
	Inguinal	7 (3)	9 (5)
	Parotic	88 (37)	50 (27)
In-transit/satellite or skin metastases		36 (15)	10 (5)
Distant organs		2 (1)	0 (0)

^a^48 metastases at baseline were excluded from the discovery NCC dataset

^b^Includes glandula submandibularis (n = 1)

^c^Includes lymphnode in upper arm (n = 1)

In both cohorts, metastases were most frequently first detected in the lymph nodes. In the discovery NCC dataset, 84% of patients with metastasis during follow-up had lymph node involvement at first diagnosis, most commonly in the parotid gland (37%) or cervical lymph nodes (33%); only two patients (1%) presented with distant metastasis. Similarly, in the validation NCC dataset, 84% of patients developed metastasis during follow-up and 16% had metastasis at baseline. Lymph node metastases were also predominant (90%), with the cervical lymph nodes being the most frequent initial site (52%). No patients in the validation NCC dataset had distant metastasis at first diagnosis (Table 5).

## Discussion

The D-SQUAME study provides a comprehensive and practical design for investigating prognostic factors in CSCC based on population-based data by addressing the low event rate with an efficient study design. It includes two large nationwide cohorts with long-term follow-up and a high number of metastatic events. The use of the linkage of nationwide databases and a nested case–control design enabled to overcome major challenges in prognostic CSCC research. By combining clinical, imaging, and molecular data from these cohorts, the study enables the identification and validation of novel prognostic factors for metastasis in CSCC.

The D-SQUAME study was designed to address the variability in clinical practice that complicates CSCC research. By including both biopsies (punch and shaves) and excisions (WLE and MSS) as performed in the Netherlands, the study captures real-world diversity in sample types and increases the likelihood that findings and biomarker performance will remain robust across sample procedures, making them applicable to routine clinical practice.

### Existing CSCC datasets

Until recently, most CSCC studies were single-center studies and therefore limited in size and generalizability [26, 30–32]. In recent years, several initiatives have started to expand available data by developing large multicenter datasets. One of the largest multinational datasets of CSCC patients was presented by Hallak et al. [33], reflecting a substantial effort to collect data from a total of 23,166 CSCCs across 12 centers in three countries: the United States (10 centers), Spain (1 center), and Brazil (1 center). While this dataset represents an important advancement in collaborative CSCC research, variations in outcome reporting and tumor characterization between centers illustrate the methodological challenges of ensuring data consistency in multicenter international studies. These differences may influence the comparability of data across sites and should be carefully addressed in pooled analyses.

Another limitation of existing datasets is the frequent reliance on data from tertiary care centers without the ability to use data from other centers [30, 33]. This may introduce selection bias, as these centers are often referral centers for high-risk CSCC cases and the culprit primary CSCC of recurrent or metastatic CSCC is frequently diagnosed earlier in time in another (general) hospital. In contrast, the D-SQUAME study is a nationwide cohort that used pathology data from all healthcare settings in the Netherlands, ranging from academic hospitals to private practices and general practitioners. This approach provides a representative sample of the entire CSCC patient population, including low- and high-risk cases.

### Molecular data on CSCC

Large-scale molecular datasets of CSCC are scarce [14]. Only a limited number of primarily small studies have explored the molecular landscape of CSCC in depth [9, 10, 34–36]. Chang and Shain performed a meta-analysis including 105 tumors from 10 different centers, providing a catalog of driver genes in CSCC [10]. Bencomo et al. [9] performed a meta-analysis of whole-transcriptome data of 306 samples representing different stages in progression from normal skin to CSCC (147 CSSCs). Few studies have investigated transcriptomic changes associated with disease progression [37–39]. In addition, Ji et al. [34] performed spatial transcriptomics on 6 CSCC samples. Most available data lack detailed clinical and pathological information.

In parallel with limited genomic data, little is known about the immune microenvironment in CSCC [35, 36]. Despite the role of immune status in skin cancer development [40, 41] and the clinical success of immune checkpoint inhibitors [42, 52], no large-scale studies have yet characterized the immune landscape of CSCC.

### Imaging data on CSCC

Histologic evaluation of H&E-stained slides remains the diagnostic standard for CSCC and is routinely used to assess high-risk features, including depth of invasion, perineural invasion (PNI), and degree of differentiation. However, their prognostic values are limited by intra- and inter-observer variability [42]. Machine learning and artificial intelligence have shown potential for analyzing H&E images to identify features associated with poor outcomes. However, only a limited number of studies have explored this approach in CSCC [43–46]. These studies remain constrained by small sample sizes and a low number of outcome events. Importantly, they rarely combine high-quality hematoxylin and eosin–stained whole slide images with comprehensive clinical data, which is essential for robust model development and validation [47, 48]. The D-SQUAME study will support this effort by expanding the availability of H&E images for model development and validation.

### Strengths and limitations

The strengths of this study include a nationwide NCC design, risk-based matching, dual validation cohorts, and a unique multimodal dataset, enabling robust biomarker discovery and reliable absolute risk prediction. A major strength of this study is that we collected a nationwide cohort and performed a NCC design. This design allowed for efficient collection of all patients with metastases, enhancing statistical power while preserving feasibility [12]. The NCC design supports the development of robust prediction models to estimate the absolute risk of metastasis. Moreover, this study demonstrates practical methods for identifying poor outcomes, such as metastasis in the absence of outcome registration, and for applying expensive molecular techniques in the context of relatively rare events.

Another strength of this study is the use of matching on metastatic risk based on the previously validated EMC model [13, 49]. This approach enhances our ability to identify novel prognostic factors by minimizing the influence of established clinical variables, which might otherwise largely explain the observed molecular associations. Importantly, we performed loose matching to reduce the chances of missing relevant biomarkers that are correlated with the matching variables.

We selected two types of controls in the validation cohort (i.e. randomly selected controls and risk-matched controls), resulting in two types of validation datasets. The matched NCC dataset, enables validation of all analytic steps applied in the discovery cohort under similar conditions. The NCC dataset with randomly selected controls allows assessment of the biomarker or prediction model's performance in a cohort that is more representative of the CSCC patient population. This NCC dataset with randomly selected controls also enables exploration of relevant thresholds for model application for specific clinical utilities such as adjuvant radiotherapy or immunotherapy.

Lastly, the D-SQUAME study presents a unique opportunity to integrate multiple data modalities. While some studies have explored predictive models that combine genomic and imaging data, a recent review identified only nine that applied such multimodal approaches to predict poor outcomes in cancer [50]. The current study not only provides a dataset suitable for the development of integrated prognostic models but also includes an independent cohort for validation.

Limitations of our study include the absence of routinely registered clinical data, reliance on older FFPE material and variability in sample processing across pathology laboratories. During the study period, clinical outcomes for CSCC were not recorded in the NCR. Therefore, the D-SQUAME study relied on a rule-based algorithm to identify cases with metastasis from pathology reports [21]. Recently, we established the Dutch Keratinocyte Cancer Collaborative (DKCC) study, which is the first nationwide database for all types of advanced CSCC (i.e. locally advanced CSCC, recurrent and metastatic CSCC) [23]. The DKCC database captures a broader range of outcomes using detailed clinical data sources.

While the use of cancer registry data enabled the inclusion of patients with long-term follow-up, it also resulted in methodological challenges due to the use of old FFPE material. In addition, these samples were originally processed in various external pathology laboratories, and therefore initial handling and storage conditions differed between different pathology labs and over time, which may have an impact on the tissue integrity. The adapted laboratory techniques that were applied to overcome these challenges for molecular analyses will be described in detail in future manuscripts.

## Conclusion

The D-SQUAME study is a nationwide study on metastatic risk prediction of CSCC with a comprehensive and practical design that allows large-scale sample collection. This design enables research on multimodal data including clinical data, imaging and multi-omics data. As a population-based study, D-SQUAME includes the full range of CSCC patients, not only those at high risk of metastasis. In doing so, it fills major gaps in current knowledge and provides an important framework for future prognostic cancer research. The nested case–control design supports the development of reliable models to predict the absolute risk of metastasis. It offers practical strategies for identifying metastasis when outcome data are not routinely recorded and for applying costly molecular analyses in prediction modelling studies with both nationwide data and rare outcomes.

## Supplementary Information

Below is the link to the electronic supplementary material.

Supplementary file1 (DOCX 31 KB)
