# Supplementary material for: The Dutch cutaneous squamous cell carcinoma and metastasis (D-SQUAME) study: a nationwide discovery cohort and nationwide validation cohort with nested case–control designs for risk prediction modeling
Source: Eur J Epidemiol. 2026 Mar 12;41(5):665–80. doi: 10.1007/s10654-026-01384-4 (PMC13332985; doi:10.1007/s10654-026-01384-4)
Supplement: Supplementary file 1 — Supplementary file1 (DOCX 31 KB) [file 10654_2026_1384_MOESM1_ESM.docx]

# Supplementary material

The Dutch Cutaneous Squamous Cell Carcinoma and Metastasis (D-SQUAME) Study: a nationwide discovery and nationwide validation cohort with nested case-control designs for risk prediction modeling

## **S1. Variables requested from the Netherlands Cancer Registry (NCR)**

Needed NCR variables of all patients with first primary CSCC in 2007-2009 and 2017-2019:

- Patient characteristics:
  - Unique patient identifier
  - Unique tumor identifier
  - Sex
  - Age at diagnosis
  - Vital status [censor data = date latest update of the data]
  - Follow up time in days [censor data = date latest update of the data]

- Tumor characteristics of the registered first primary cSCC:
  - Year of incidence
  - Topography (localization)
  - Lateralization
  - Morphology
  - Histology (including ‘morfologie’, ‘tumorgedrag’, ‘multifocaliteit’)
  - Differentiation (‘differentiatiegraad’)
  - Multifocaliteit
  - Stage
  - cTNM
  - pTNM
- Other hematological malignancy before and after first cSCC
  - Type of hematological malignancy:
    - Hodgkin lymphoma
    - B-CLL / small cell B-cell lymphoma
    - Indolent non-Hodgkin lymphoma
    - Aggressive non-Hodgkin lymphoma
    - Plasma cell tumors
    - Mature T-cell and NK-cell tumors (excluding cutaneous lymphomas)
    - Cutaneous lymphomas
    - Lymphoblastic leukemia/lymphoma
    - Acute myeloid leukemia
    - Unspecified and biphenotypic leukemia
    - Myeloproliferative disorders
  - Number of days between first CSCC and incidence date of hematological malignancy

## **S2. Codelists (supplementary Excel file)**

## **S3. Sample processing protocol**

### Batch preparation

Formalin-fixed paraffin-embedded (FFPE) tumour blocks were sent by participating pathology laboratories to the central pathology unit at the Erasmus University Medical Centre (Erasmus MC). Upon arrival, each delivery was checked to confirm that the received blocks matched the requested specimens. When multiple blocks were provided, the selection followed these criteria:

- Blocks taken from the borders of the excision (head or tail) were excluded.
- The selected block had to contain visible tumour tissue, as described in the microscopy section of the pathology report.
- If the microscopy description was unclear or unavailable, the block was macroscopically inspected, and the block containing the largest amount of tissue was selected.

After selection, blocks were organized into batches for sectioning. For the development cohort, each batch consisted of 25 case-control pairs. For the validation cohort, each batch consisted of 48 FFPE blocks, including 16 cases, 16 random controls, and 16 matched controls.

Sectioning followed a first-in, first-cut approach, meaning that blocks were processed in the order they were received. All blocks were labelled according to the established labelling protocol. If any label detached during handling, it was checked and replaced before further processing. To enable correction for batch effects and the potential effects of different pathology labs in sequencing analyses, blocks from the same laboratory were distributed across different batches.

Completed batches were then sent for sectioning according to the scheme described in sTable 1.

One participating pathology laboratory preferred not to send FFPE blocks and instead prepared and sent scraped RNA and unstained slides to Erasmus MC. For these cases, the laboratory received the cutting procedure and a list of study codes from the research team to ensure that all slides were correctly labelled before shipment. Upon arrival at Erasmus MC, the slides were checked and stored or subsequently stained. When slides were received instead of FFPE blocks, this information was recorded in the study data files.

**Cutting and Staining Procedures**

All slides and Eppendorf tubes were labelled with a unique study code according to the labelling protocol. Slides were cut at a thickness of 4 µm. The processing of slides followed the following specifications:

- Slides for RNA isolation: Whole slides were scraped and collected in an Eppendorf tube in order to enable transcriptomics analyses of both the tumour and the surrounding tumor microenvironment. Details on RNA isolation will be described in the respective manuscript(s).
- Slides for DNA isolation: slides were kept blanco and stored in slide boxes for later use. At the time of DNAseq H&E staining was performed and macrodissection was applied. DNA was isolated from both tumor and normal from the adjacent skin. Details on DNA isolation will be described in the respective manuscript(s).
- Slides for immunohistochemistry (IHC): sections were mounted on adhesive glass slides, dipped in paraffin to preserve tissue integrity for future analyses. Any excess paraffin was carefully removed.
- Slides for haematoxylin and eosin (H&E) staining: the first section of each block was stained with H&E for quality control and histopathological evaluation.
- Blank slides: after every set of five sections, one blank slide was cut. These blank slides were reserved for potential additional analyses, such as repeated H&E staining or other future applications.

sTable 1: Overview of sectioning and processing of FFPE tumor samples

| **Slide number** | **Label** | **Glass or Eppendorf tube** | **Staining** | **Processing** |
| --- | --- | --- | --- | --- |
| 1 | H&E | Glass | H&E | Digitalization |
| 2 | RNA | Eppendorf tube | Blanco | - |
| 3 | DNA | Glass | Blanco | - |
| 4 | RNA | Eppendorf tube | Blanco | - |
| 5 | DNA | Glass | Blanco | - |
| 6 | Blanco* | Glass | Blanco |  |
| 7 | IHC | Glass (Adhesive) | Blanco | dip paraffin |
| 8 | RNA | Eppendorf tube | Blanco | - |
| 9 | DNA | Glass | Blanco | - |
| 10 | RNA | Eppendorf tube | Blanco | - |
| 11 | DNA | Glass | Blanco | - |
| 12 | Blanco* | Glass | Blanco |  |
| 13 | IHC | Glass (Adhesive) | Blanco | dip paraffin |
| 14 | RNA | Eppendorf tube | Blanco | - |
| 15 | DNA | Glass | Blanco | - |
| 16 | RNA | Eppendorf tube | Blanco | - |
| 17 | DNA | Glass | Blanco | - |
| 18 | Blanco* | Glass | Blanco |  |
| 19 | IHC | Glass (Adhesive) | Blanco | dip paraffin |
| 20 | RNA | Eppendorf tube | Blanco | - |
| 21 | DNA | Glass | Blanco | - |
| 22 | RNA | Eppendorf tube | Blanco | - |
| 23 | DNA | Glass | Blanco | - |
| 24 | Blanco* | Glass | Blanco |  |
| 25 | IHC | Glass (Adhesive) | Blanco | dip paraffin |
| 26 | RNA | Eppendorf tube | Blanco | - |
| 27 | DNA | Glass | Blanco | - |
| 28 | RNA | Eppendorf tube | Blanco | - |
| 29 | DNA | Glass | Blanco | - |
| 30 | RNA | Eppendorf tube | Blanco | - |
| 31 | RNA | Eppendorf tube | Blanco | - |
| 32 | RNA | Eppendorf tube | Blanco | - |

*Blanco slides were only collected for the validation cohort. Abbreviations: H&E: haematoxylin and eosin, IH: Immunohistochemistry

## **S4. Histopathological reassessment**

We defined the definitions of the refined pathology variables based on existing literature and standard practices. These definitions were reviewed with a group of pathologists to ensure consistent use across the study (sTable 2). FFPE tissue blocks from excision specimens of all cases and controls were collected from pathology archives. New H&E slides were prepared, scanned with a Nanozoomer S360MD Slide Scanner, and reviewed digitally in NDP.view2. Each slide was assessed by a pathologist who was blinded to patient outcomes and the pathology report. Only information about tumor location was shared to help determine invasion depth. Each pathologist worked with a research assistant who had access to the full pathology report. When major differences were found between the report and the new slide, the findings were discussed within the pathology team to avoid missing important details.

When excision specimens could not be reassessed, we reviewed the biopsy samples (when available). Biopsies were mainly used to assess differentiation grade and, when possible, perineural or lymphovascular invasion. Features that require the full invasive border could not be reliably scored on biopsy specimens, and this limitation was noted during scoring. Excision specimens were preferred whenever possible to allow full histopathological evaluation. Features that could not be reassessed were reported as missing.

sTable 2. Histopathological variables used for revising hematoxylin and eosin slides of cases and controls

| **Variable** | **Definition** | **Literature** |
| --- | --- | --- |
| Depth of invasion | The Depth of invasion (DOI) is defined as the measurement from the granular layer of the adjacent uninvolved dermis to the base of the tumor (deepest point of invasion). The DOI was measured in millimeters and rounded to whole numbers | (1-3) |
| Tissue involvement | Indicates the skin layer where the deepest point of invasion is observed. This categorical variable was classified as dermis, subcutaneous fat, or beyond subcutaneous fat. | (4-7) |
| Differentiation grade (Broder) | We categorized cells based on the proportion of poorly differentiated component present. If less than 25% of the cells are poorly differentiated, the tissue is classified as well-differentiated. When 25% to 75% of the cells are poorly differentiated, the tissue is considered moderately differentiated. Finally, if more than 75% of the cells are poorly differentiated, the tissue is classified as poorly differentiated. | (6-8), |
| Differentiation grade (worst pattern) | Classified according to its most poorly differentiated region, irrespective of the percentage present.  *Only available for the validation cohort.* | (9) |
| Perineural invasion | Defined as tumor cells in close proximity to, encircling, or infiltrating nerve fibers. If PNI was present, the diameter of the invaded nerve was measured in millimeters, referring to the widest area of the nerve itself affected by tumor cells., | (2, 4, 6, 10) |
| Lymphovascular invasion | Defined as the presence of tumor cells within lymphatic channels or blood vessels | (6, 7, 11) |
| Peritumoral infiltrate | Defined as the presence of immune cells surrounding the tumor, and classified it into three categories: absent/mild, moderate, and abundant.  Moderate peritumoral infiltrate was characterized by larger patches or a continuous thin band of cells encircling the tumor. Abundant peritumoral infiltrate was defined by a continuous thick band of cells surrounding the tumor, indicating a more robust immune response or tissue reaction adjacent to the tumor. | (12) |
| Solar elastosis | The degree of solar elastosis was categorized it into three levels: absent, moderate, and extensive. If solar elastosis was absent, no abnormal elastic tissue due to sun exposure was detected. Moderate solar elastosis was noted when there was a noticeable amount of abnormal elastic fibers present in an inconsistent, more spread out pattern on the HE slide. | (13-15) |
| Morphological subtype | We adopted the definitions outlined by the World Health Organization (WHO) Classification of Tumors. The WHO describes “acantholytic CSCC”, “clear cell CSCC”, “adenosquamous CSCC” and “spindle cell CSCC” | (16) |
| Desmoplastic CSCC | Characterized by the infiltration of atypical squamous epithelial cells forming single cell-strands at the invasive border within a distinct sclerotic stromal reaction. Notably, the desmoplastic stromal response should be more prominent than the tumor strands. To classify a tumor as desmoplastic, at least one-third of the tumor must exhibit desmoplastic features | (17) |
| Follicular CSCC (fSCC) | Defined using the following histological criteria:  • Abrupt connections of the tumour with the epidermis at the site of follicular infundibula.  • Presence of infundibular and/or tricholemmal differentiation.  • Malignant cytological and/or architectural features.  • Absence of bowenoid dysplasia or features of KA (including uncertain KA-like or KA with malignant transformation).  *Only available for the validation cohort* | (18, 19) |
| Mitotic rate | The number of mitotic cells is counted in one high power field (1 HPF) at the invasive border of the tumor. Several HPF were scanned at the invasive border to be able to find a representative HPF. A HPF was defined as 40 times enlargement on a digital HE slide assessed with NDP Viewer 2.  *Only available for the development cohort.* | (20, 21) |
| Tumor budding | Defined as the presence of one isolated single cell or small cell clusters of 1-5 cells scattered in the stroma ahead of the invasive tumor front. The area with the highest budding was selected and the number of buds was counted in 1 HPF. In case there were multiple fields of tumor buds, the number was expressed as the mean number of tumor buds in five adjacent HPFs.  *Only available for the development cohort.* | (22-24) |

**References**

1. Edge SB, American Joint Committee on Cancer ACS. AJCC cancer staging handbook: from the AJCC cancer staging manual: Springer; 2010.

2. Zakhem GA, Pulavarty AN, Carucci J, Stevenson ML. Association of Patient Risk Factors, Tumor Characteristics, and Treatment Modality With Poor Outcomes in Primary Cutaneous Squamous Cell Carcinoma: A Systematic Review and Meta-analysis. JAMA Dermatology. 2023;159(2):160-71. doi:10.1001/jamadermatol.2022.5508

3. Stratigos AJ, Garbe C, Dessinioti C, et al. European consensus-based interdisciplinary guideline for invasive cutaneous squamous cell carcinoma. Part 1: Diagnostics and prevention–Update 2023. European Journal of Cancer. 2023;193:113251. doi:https://doi.org/10.1016/j.ejca.2023.113251

4. Thompson AK, Kelley BF, Prokop LJ, Murad MH, Baum CL. Risk Factors for Cutaneous Squamous Cell Carcinoma Recurrence, Metastasis, and Disease-Specific Death: A Systematic Review and Meta-analysis. JAMA Dermatology. 2016;152(4):419-28. doi:10.1001/jamadermatol.2015.4994

5. Ruiz ES, Karia PS, Besaw R, Schmults CD. Performance of the American Joint Committee on Cancer Staging Manual, 8th Edition vs the Brigham and Women's Hospital Tumor Classification System for Cutaneous Squamous Cell Carcinoma. JAMA Dermatol. 2019;155(7):819-25.

6. Tokez S, Venables ZC, Hollestein LM, et al. Risk factors for metastatic cutaneous squamous cell carcinoma: Refinement and replication based on 2 nationwide nested case-control studies. J Am Acad Dermatol. 2022;87(1):64-71. doi:10.1016/j.jaad.2022.02.056

7. Rentroia-Pacheco B, Tokez S, Bramer EM, et al. Personalised decision making to predict absolute metastatic risk in cutaneous squamous cell carcinoma: development and validation of a clinico-pathological model. EClinicalMedicine. 2023;63:102150. doi:10.1016/j.eclinm.2023.102150

8. Broders AC. SQUAMOUS-CELL EPITHELIOMA OF THE SKIN: A STUDY OF 256 CASES. Ann Surg. 1921;73(2):141-60. doi:10.1097/00000658-192102000-00001

9. Slater D, Barrett P. Dataset for Histopathological Reporting of Primary Invasive Cutaneous Squamous Cell Carcinoma and Regional Lymph Nodes. London, UK: The Royal College of Pathologists2019.

10. Ross AS, Miller Whalen F, Elenitsas R, Xu X, Troxel AB, Schmults CD. Diameter of involved nerves predicts outcomes in cutaneous squamous cell carcinoma with perineural invasion: An investigator-blinded retrospective cohort study. Dermatol Surg. 2009;35(12):1859-66. doi:10.1111/j.1524-4725.2009.01354.x

11. Farah M, Milton DR, Gross ND, et al. Histopathologic features predictive of metastasis and survival in 230 patients with cutaneous squamous cell carcinoma of the head and neck and non-head and neck locations: a single-center retrospective study. Journal of the European Academy of Dermatology and Venereology. 2022;36(8):1246-55. doi:https://doi.org/10.1111/jdv.18147

12. Quaedvlieg PJ, Creytens DH, Epping GG, et al. Histopathological characteristics of metastasizing squamous cell carcinoma of the skin and lips. Histopathology. 2006;49(3):256-64.

13. Berwick M, Reiner AS, Paine S, et al. Sun exposure and melanoma survival: a GEM study. Cancer Epidemiol Biomarkers Prev. 2014;23(10):2145-52. doi:10.1158/1055-9965.Epi-14-0431

14. Chang H-C, Cheng HY, Lee LT-J. Solar elastosis and melanoma-specific survival: A systematic review and meta-analysis. Journal of the European Academy of Dermatology and Venereology. 2023;37(5):951-3. doi:https://doi.org/10.1111/jdv.18684

15. Mishra K, Barnhill RL, Paddock LE, Fine JA, Berwick M. Histopathologic variables differentially affect melanoma survival by age at diagnosis. Pigment Cell Melanoma Res. 2019;32(4):593-600. doi:10.1111/pcmr.12770

16. International Agency for Research on C. WHO Classification of Tumours. Lyon, France: International Agency for Research on Cancer; 2020.

17. Breuninger H, Schaumburg-Lever G, Holzschuh J, Horny HP. Desmoplastic squamous cell carcinoma of skin and vermilion surface. A highly malignant subtype of skin cancer. CANCER. 1997;79(5):915-9. doi:10.1002/(sici)1097-0142(19970301)79:5<915::Aid-cncr7>3.0.Co;2-a

18. Carr RA, Taibjee SM, Turnbull N, Attili S. Follicular squamous cell carcinoma is an under-recognised common skin tumour. Diagnostic Histopathology. 2014;20(7):289-96. doi:https://doi.org/10.1016/j.mpdhp.2014.05.003

19. Carr RA, Wiggins J, Slater DN. Follicular (Infundibular-Tricholemmal) Squamous Cell Carcinoma: A New WHO Entity. Clinicopathological Features in 103 Cases, Including Follow-Up and Implications for Patient Management. Am J Dermatopathol. 2024;46(7):416-32.

20. Mandalà M, Galli F, Cattaneo L, et al. Mitotic rate correlates with sentinel lymph node status and outcome in cutaneous melanoma greater than 1 millimeter in thickness: A multi-institutional study of 1524 cases. J Am Acad Dermatol. 2017;76(2):264-73 e2. doi:https://doi.org/10.1016/j.jaad.2016.08.066

21. Thompson JF, Soong SJ, Balch CM, et al. Prognostic significance of mitotic rate in localized primary cutaneous melanoma: an analysis of patients in the multi-institutional American Joint Committee on Cancer melanoma staging database. J Clin Oncol. 2011;29(16):2199-205. doi:https://doi.org/10.1200/JCO.2010.31.5812

22. Fujimoto M, Yamamoto Y, Takai T, et al. Tumor Budding Is an Objective High-risk Factor Associated With Metastasis and Poor Clinical Prognosis in Cutaneous Squamous Cell Carcinoma Sized <4 cm. The American Journal of Surgical Pathology. 2019;43(7):975-83. doi:10.1097/pas.0000000000001284

23. Gonzalez-Guerrero M, Martínez-Camblor P, Vivanco B, et al. The adverse prognostic effect of tumor budding on the evolution of cutaneous head and neck squamous cell carcinoma. J Am Acad Dermatol. 2017;76(6):1139-45. doi:https://doi.org/10.1016/j.jaad.2017.01.015

24. Kanitakis J, Karayannopoulou G. Prognostic significance of tumor budding in cutaneous squamous cell carcinoma. Journal of the American Academy of Dermatology. 2018;79(1):e5. doi:https://doi.org/10.1016/j.jaad.2017.07.060
